# Combination of CALR and PDIA3 is a potential prognostic biomarker for non-small cell lung cancer

**DOI:** 10.18632/oncotarget.18547

**Published:** 2017-06-16

**Authors:** Ke Wang, Hao Li, Ruo Chen, Yang Zhang, Xiu-Xuan Sun, Wan Huang, Huijie Bian, Zhi-Nan Chen

**Affiliations:** ^1^ Department of Cell Biology, National Translational Science Center for Molecular Medicine, Fourth Military Medical University, Xi'an, P.R. China; ^2^ Department of Cell Biology, College of Life Science and Technology, Jinan University, Guangzhou, P.R. China

**Keywords:** CALR, PDIA3, non-small cell lung cancer, biomarker, proteomics

## Abstract

Proteomic-based approaches for biomarker discovery are promising strategies used in cancer research. In this study, we performed quantitative proteomic analysis on 16 paired samples of non-small cell lung cancer (NSCLC) and adjacent non-tumor lung tissues using label-free quantitative proteomics and liquid chromatography-tandem mass spectrometry/mass spectrometry (LC-MS/MS) to identify differentially expressed proteins. A total of 91 proteins were differentially expressed in NSCLC compared with adjacent non-tumor lung tissues among 4047 identified proteins (fold change > 1.5 or < 0.67, *P* < 0.05). Gene ontology (GO) analysis, Kyoto encyclopedia of genes and genomes (KEGG) pathway analysis and ingenuity pathway analysis (IPA) of 91 dysregulated proteins showed that they were related to the cancer-associated biological processes. We confirmed that the candidate proteins, calreticulin (CALR) and protein disulfide isomerase family A member 3 (PDIA3) were overexpressed in NSCLC by real-time PCR using 20 paired samples and western blot using 5 paired samples. PDIA3 expression was highly associated with CALR expression (Spearman *r* = 0.345, *P* = 0.001) and they were co-localized and interacted with each other in A549 and H460 cells. Moreover, survival analysis performed in tissue microarray with 88 samples indicated that low expression of both CALR and PDIA3 in NSCLC was positively associated with poor overall survival. Combination of CALR and PDIA3 might serve as an efficient biomarker and improved the prediction of NSCLC prognosis significantly (*P* = 0.023). Our results collectively provide a potential biomarker dataset for NSCLC prognosis, especially the prognostic value of combined expression of CALR and PDIA3.

## INTRODUCTION

Lung cancer is the most common incident cancer and the leading cause of the cancer death in China [1]. The incidence and mortality of lung cancer in China have been growing rapidly in recent years, leading to high social and economic burdens [2]. The estimated incidence and mortality of lung cancer in 2015 are higher in men and urban areas than those in women and rural areas [3]. The majority of lung cancer is non-small cell lung cancer (NSCLC), which includes four histologic types: squamous cell carcinoma, adenocarcinoma, large cell carcinoma and mixed histologies [4, 5]. One of most important reasons behind the high incidence and the low 5-year survival rate of NSCLC in China is related to little opportunity for early prediction and diagnosis. The application of biomarkers has a potential to help in prediction, diagnosis and evaluating the role of related therapies [6]. Hence, an increasing number of specific biomarkers are needed to improve patient management and increase patient survival.

Proteomics-based technologies are utilized in various capacities for different research settings such as detection of various diagnostic markers, candidates for vaccine production, understanding pathogenicity mechanisms, alteration of expression patterns in response to different signals and interpretation of functional protein pathways in different diseases [7]. In recent years, proteomic technologies have been widely used in the biomarker discovery of cancer. The identification of molecular markers for early prediction and diagnosis of cancers will have a great effect in improving patient management.

In this study, we performed a quantitative proteomic analysis using label-free quantitative proteomics and LC-MS/MS to identify differentially expressed proteins between NSCLC and paired adjacent non-tumor lung tissues and provided a potential biomarker dataset for NSCLC prognosis. Two candidate proteins, CALR and PDIA3 were validated using real-time PCR, western blot and immunohistochemistry analysis. Further analysis evaluated the prognostic value of CALR and PDIA3 in NSCLC.

## RESULTS

### Identification of differentially expressed proteins in sixteen paired NSCLC and adjacent non-tumor lung tissues

Sixteen paired samples of NSCLC and adjacent non-tumor lung tissues were performed by trypsin digestion and subject to analysis with mass spectrometer. The result showed that a total of 91 out of 4047 identified proteins were differentially expressed with 71 proteins increased in the NSCLC tissues and 20 proteins increased in the adjacent non-tumor lung tissues (*P* < 0.05, Supplementary Table 1).

### GO analysis

**Figure 1:**
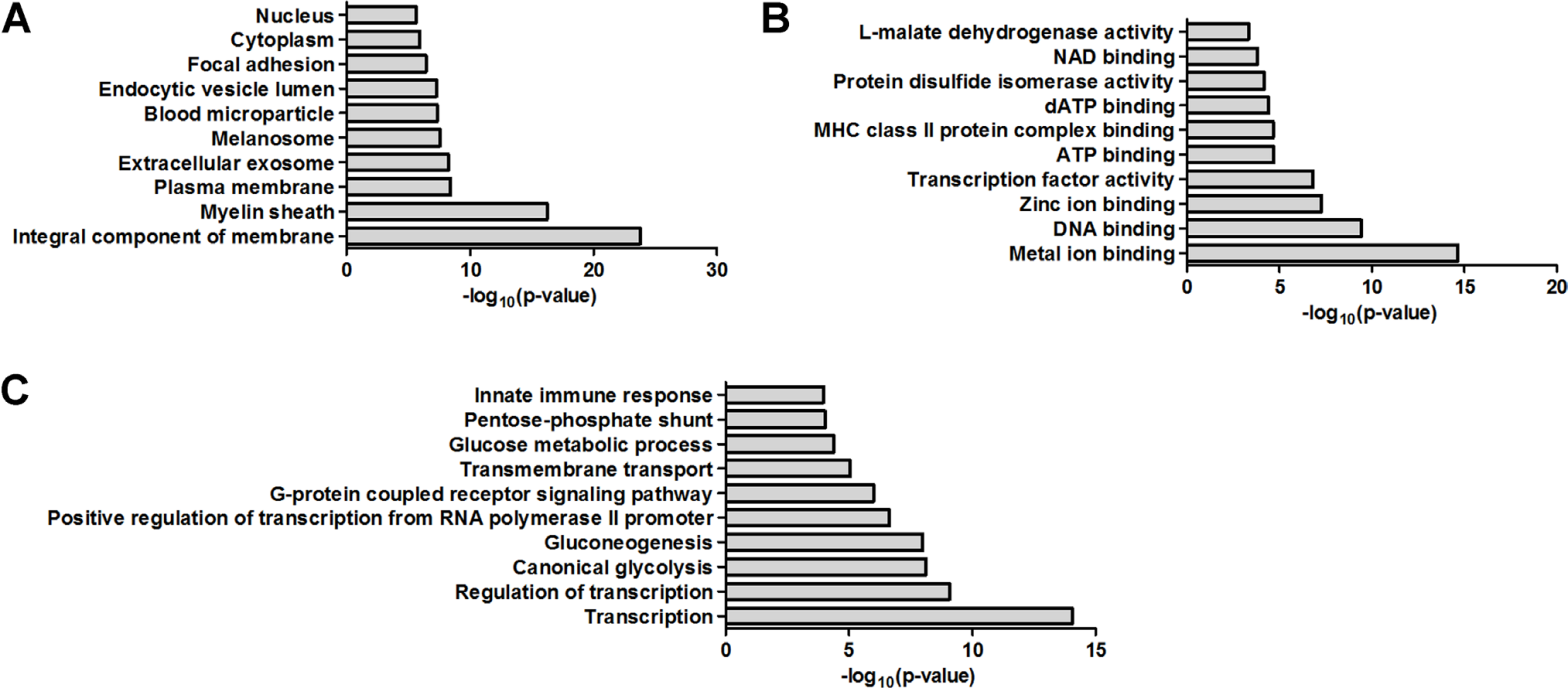
Bioinformatics analysis of the dysregulated proteins by GO analysis (**A**) Cellular component. (**B**) Molecular function. (**C**) Biological process.

The functional interpretation of 91 dysregulated proteins was annotated using the GO via enrichment analysis. In cellular component, the top ten GO terms were integral component of membrane, myelin sheath, plasma membrane, extracellular exosome, melanosome, blood microparticle, endocytic vesicle lumen, focal adhesion, cytoplasm and nucleus (Figure 1A). In molecular function, the top ten GO terms were metal ion binding, DNA binding, zinc ion binding, transcription factor activity, ATP binding, MHC class II protein complex binding, dATP binding, protein disulfide isomerase activity, NAD binding and L-malate dehydrogenase activity (Figure 1B). In biological process, the top ten GO terms were transcription, regulation of transcription, canonical glycolysis, gluconeogenesis, positive regulation of transcription from RNA polymerase II promoter, G-protein coupled receptor signaling pathway, transmembrane transport, glucose metabolic process, pentose-phosphate shunt and innate immune response (Figure 1C).

### KEGG pathway analysis

**Figure 2:**
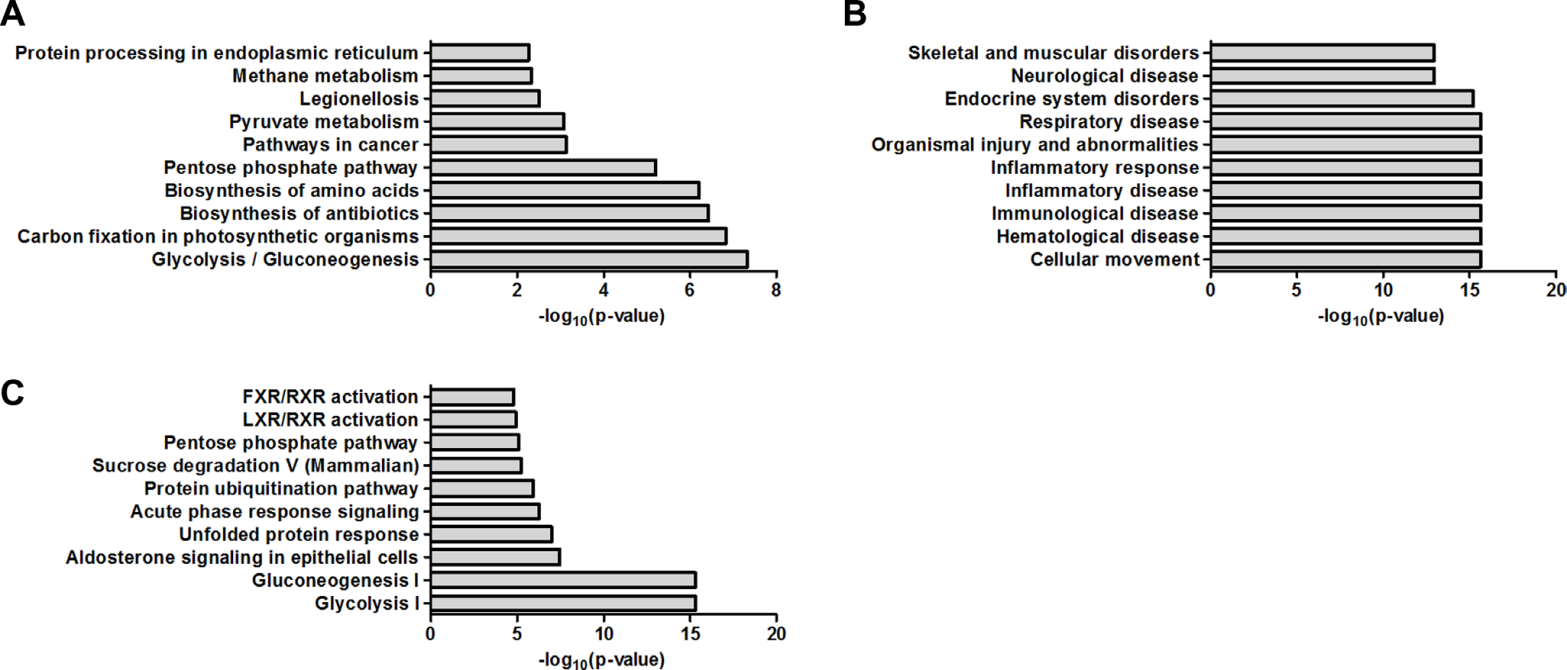
Bioinformatics analysis of the dysregulated proteins by KEGG pathway analysis and IPA (**A**) Biochemical pathways and signal transduction pathways by KEGG pathway analysis. (**B**) Disease/functions by IPA. (**C**) Canonical pathways by IPA.

KEGG pathway is a database for systematic analysis of molecular interaction and reaction networks for metabolism, genetic information processing, environmental information processing, cellular processes, organismal systems and human diseases. By KEGG basic pathway mapping tool, 91 dysregulated proteins were revealed to be related with pathways including glycolysis/gluconeogenesis, carbon fixation in photosynthetic organisms, biosynthesis of antibiotics, biosynthesis of amino acids, pentose phosphate pathway, pathways in cancer, pyruvate metabolism, legionellosis, methane metabolism and protein processing in endoplasmic reticulum (Figure 2A).

### IPA

The 91 dysregulated proteins were analyzed with IPA which contains disease/functions, canonical pathways and network analysis. The top ten terms for disease/functions were cellular movement, hematological disease, immunological disease, inflammatory disease, inflammatory response, organismal injury and abnormalities, respiratory disease, endocrine system disorders, neurological disease and skeletal and muscular disorders (Figure 2B). The top ten terms for canonical pathways were glycolysis I, gluconeogenesis I, aldosterone signaling in epithelial cells, unfolded protein response, acute phase response signaling, protein ubiquitination pathway, sucrose degradation V (Mammalian), pentose phosphate pathway, LXR/RXR activation and FXR/RXR activation (Figure 2C). The network analysis were carried out and revealed six main interaction networks including carbohydrate metabolism/cellular movement/hematological disease, post-translational modification/protein folding/nucleic acid metabolism, endocrine system disorders/organismal injury and abnormalities/neurological disease, cancer/gastrointestinal disease/hepatic system disease, connective tissue disorders/developmental disorder/hereditary disorder and carbohydrate metabolism/nucleic acid metabolism/small molecule biochemistry (Supplementary Figures 1–6).

### Validation of dysregulated protein (CALR and PDIA3) expression

**Figure 3:**
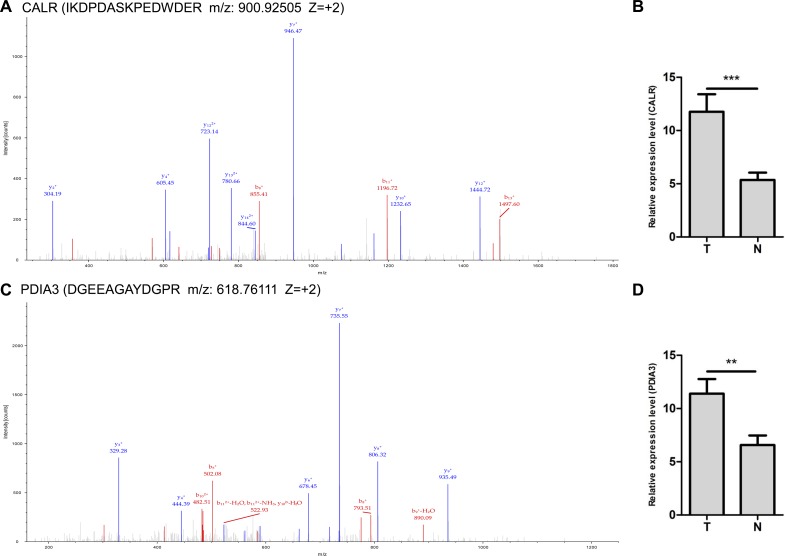
LC-MS/MS spectra used for the identification and quantitation of CALR and PDIA3 (**A**) The sequence IKDPDASKPEDWDER allowed the identification of CALR. (**B**) IBAQ got from MaxQuant provided the relative quantitation of CALR from NSCLC (T) and adjacent non-tumor lung tissues (N). ^***^P < 0.001. (**C**) The sequence DGEEAGAYDGPR allowed the identification of PDIA3. (**D**) IBAQ got from MaxQuant provided the relative quantitation of PDIA3 from NSCLC (T) and adjacent non-tumor lung tissues (N). ^**^P < 0.01.

**Figure 4:**
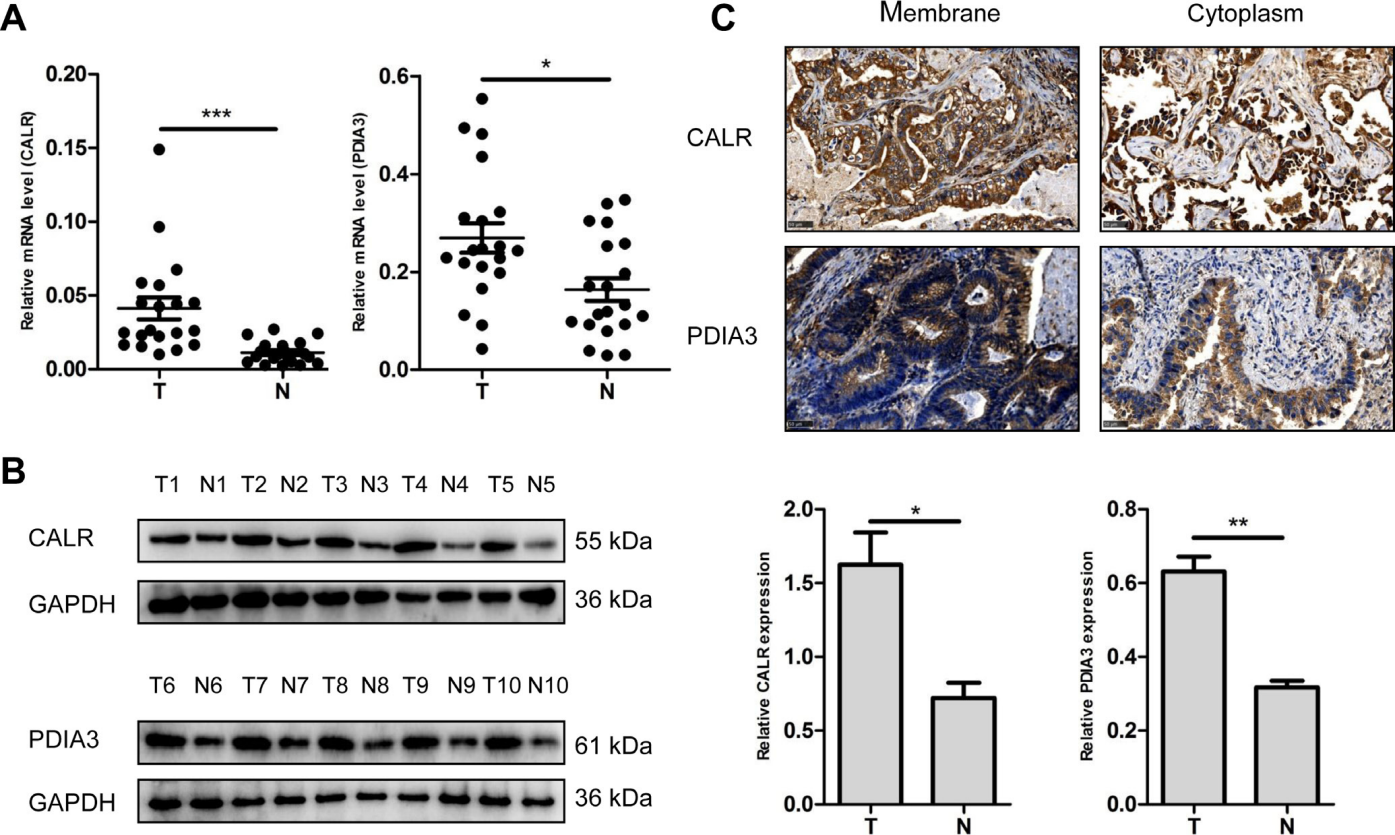
Validation of dysregulated CALR and PDIA3 expressions (**A**) Detection of CALR and PDIA3 expressions in NSCLC (T) and adjacent non-tumor lung tissues (N) using real-time PCR. *n* = 20, ^*^P < 0.05, ^***^P < 0.001. (**B**) Validation of CALR and PDIA3 expressions in NSCLC (T) and adjacent non-tumor lung tissues (N) using western blot. GAPDH was used as a control. The histograms show quantification of the gray scale analysis for western blot. *n* = 5, ^*^P < 0.05, ^**^P < 0.01 (**C**) Cellular localization of CALR and PDIA3 using immunohistochemistry. Scale bar = 50 μm.

Two up-regulated proteins CALR and PDIA3 were selected as candidate molecular biomarkers to be evaluated. The sequence IKDPDASKPEDWDER allowed the identification of CALR (Figure 3A), and the sequence DGEEAGAYDGPR allowed the identification of PDIA3 (Figure 3C). LC-MS/MS-based quantitative analysis showed that CALR and PDIA3 were significantly up-regulated in NSCLC tissues relative to adjacent non-tumor lung tissues (Figure 3B and 3D). The over-expressions of CALR and PDIA3 in tumor tissues were confirmed by real-time PCR using 20 paired tissues (Figure 4A), and verified by western blot analysis (Figure 4B). Immunohistochemistry analysis of TMA displayed that CALR and PDIA3 were mainly localized in the membrane and cytoplasm of the tumor cells (Figure 4C). Both proteins were up-regulated in the tumor tissues compared with the paired adjacent non-tumor lung tissues (both *P* < 0.01, Table 1).

**Table 1:** CALR and PDIA3 expression in NSCLC (T) and adjacent non-tumor lung tissues (N)

Levels of staining	CALR		PDIA3	
	T	N	T	N
–	0	0	0	37
+	22	31	30	45
++	36	52	36	2
+++	26	1	18	0

### Correlations of CALR and PDIA3 expressions with clinicopathological parameters of patients with NSCLC

To characterize the roles of CALR and PDIA3 in NSCLC development, the relationships between the two proteins expression and clinicopathological parameters of NSCLC patients were analyzed (Table 2). The expression of CALR in NSCLC was not significantly related to the gender, age, tumor sizes, lymph node metastases, AJCC stages and pathological grades. Similar association between PDIA3 expression and these clinicopathological parameters was also observed in NSCLC.

**Table 2:** Correlations of CALR and PDIA3 expressions with clinicopathological parameters of patients with NSCLC

		CALR			PDIA3		
Parameters	n	Low	High	*P*	Low	High	*P*
		(*n* = 25)	(*n* = 63)		(*n* = 33)	(*n* = 55)	
Gender							
Male	46	12	34	0.613	20	26	0.225
Female	42	13	29		13	29	
Age (years)							
≥ 62	44	13	31	0.813	14	30	0.271
< 62	44	12	32		19	25	
Tumor sizes (cm)							
≤ 3	33	7	26	0.246	10	23	0.280
> 3	55	18	37		23	32	
Lymph node metastases							
0	37	13	24	0.233	12	25	0.403
≥ 1	51	12	39		21	30	
AJCC stages							
I	27	8	19	0.866	8	19	0.310
II + III	61	17	44		25	36	
Pathological grades							
I + I–II + II	62	19	43	0.473	25	37	0.398
II–III + III	26	6	20		8	18	

Cases of group “–” and “+” were assigned to group low. Cases of group “++” and “+++” were assigned to group high.

### Positive correlation between CALR and PDIA3 expressions in NSCLC and CALR-PDIA3 interaction

**Figure 5:**
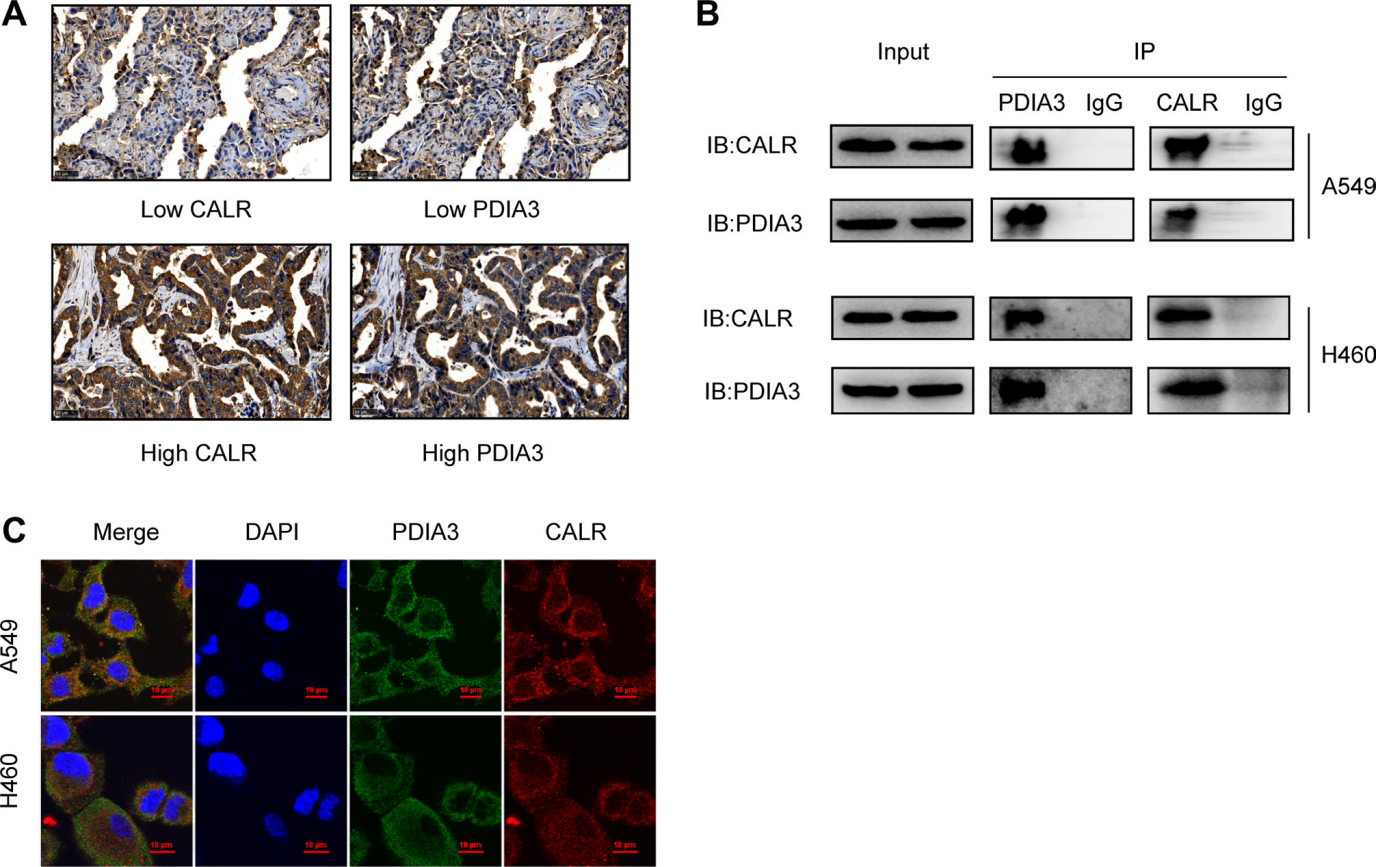
Correlation of CALR and PDIA3 in NSCLC (**A**) Co-expression of CALR and PDIA3 in patients with NSCLC detected by immunohistochemistry. Scale bar = 50 μm. (**B**) Interaction of CALR-PDIA3 in two lung cancer cell lines tested by co-IP assay. (**C**) Co-localization of CALR and PDIA3 in A549 and H460 cell lines visualized under a confocal florescence microscopy. Scale bar = 10 μm.

To investigate the association between CALR and PDIA3 expressions, immunohistochemistry analysis of 88 cases of tumor tissues from TMAs was performed and indicated that PDIA3 expression was highly associated with CALR expression (Spearman *r* = 0.345, *P* = 0.001) (Table 3). Representative images of CALR and PDIA3 expressions from serial tissue sections were shown in Figure 5A. Co-IP assay demonstrated an interaction of CALR-PDIA3 in two lung cancer cell lines, A549 and H460 (Figure 5B). The cellular co-localization of CALR and PDIA3 were illustrated by immunofluorescence staining (Figure 5C).

**Table 3:** Correlation between CALR and PDIA3 expressions in NSCLC

	CALR			Spearman’s correlation	
PDIA3	Low	High	Total	rho	*P*
Low	16	17	33		
High	9	46	55	0.345	0.001
Total	25	63	88		

Cases of group “–” and “+” were assigned to group low. Cases of group “++” and “+++” were assigned to group high.

### Expressions of CALR and PDIA3 in prognosis of patients with NSCLC

**Figure 6:**
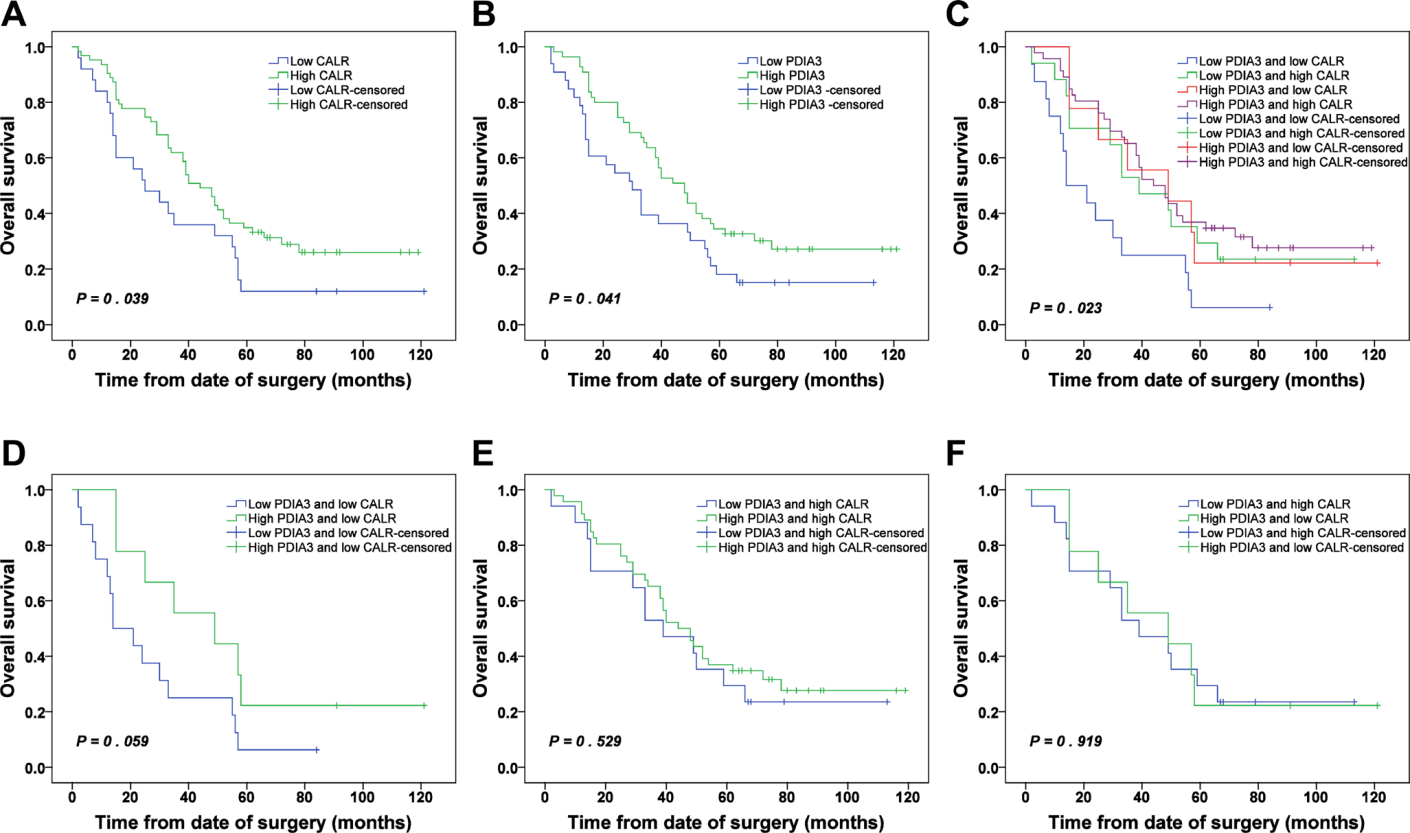
Kaplan-Meier survival analysis of CALR and PDIA3 expressions in NSCLC (**A**) OS of NSCLC patients with high and low CALR expressions. (**B**) OS of NSCLC patients with high and low PDIA3 expressions. (**C**) OS of NSCLC patients with combined expression of CALR and PDIA3. (**D**) OS of NSCLC patients with low PDIA3/low CALR and high PDIA3/low CALR. (**E**) OS of NSCLC patients with low PDIA3/high CALR and high PDIA3/high CALR. (**F**) OS of NSCLC patients with low PDIA3/high CALR and high PDIA3/low CALR.

Patients with low CALR expression and low PDIA3 expression were significantly associated with poor OS (*P* = 0.039 and *P* = 0.041, respectively) (Figure 6A and 6B). The cumulative 5-year OS rates of patients with low and high expressions of CALR were 12.0% and 34.9%, respectively. Similarly, patients with low and high expressions of PDIA3 were 18.2% and 34.5 %, respectively.

Due to a significant positive correlation between CALR and PDIA3 expressions in NSCLC (Table 3), we wonder whether the prediction of NSCLC prognosis was more accurate relying on combined expression of CALR and PDIA3 than single alone. The patients were classified into 4 subgroups: a) low expression of CALR and low expression of PDIA3; b) low expression of CALR and high expression of PDIA3; c) high expression of CALR and low expression of PDIA3; d) high expression of CALR and high expression of PDIA3. Kaplan-Meier analysis showed that a significant difference was found among 4 subgroups (*P* = 0.023). The patients with low expression of CALR and low expression of PDIA3 had the poorest prognosis, and conversely, the patients with high expression of CALR and high expression of PDIA3 had the best prognosis (Figure 6C). Whereas the survivals of patients with low CALR/high PDIA3 and high CALR/low PDIA3 were not statistically different (Figure 6F). Additionally, in the low expression CALR subgroup, the survival of patients with low expression PDIA3 or high expression PDIA3 showed no statistical difference (Figure 6D). Similarly, in the high expression CALR subgroup, the survival of patients with low expression PDIA3 or high expression PDIA3 also showed no statistical difference (Figure 6E).

Cox regression analysis was carried out to access the significance of CALR and PDIA3 in the prognosis of NSCLC. Covariates included gender, age, AJCC stages, pathological grades, CALR expression and PDIA3 expression. The results indicated that AJCC stages and CALR expression were found to be independent prognostic factors for patients with NSCLC (Table 4). However, when combined expression of CALR and PDIA3 was added to the Cox regression analysis, AJCC stages and combined expression of CALR and PDIA3 were found to be independent prognostic factors (Table 5). These results indicated that combined expression of CALR and PDIA3 was more accurate in prediction of NSCLC prognosis compared with CALR and PDIA3 expression individually.

**Table 4:** Cox multivariate analysis of the correlation between clinicopathological parameters and survival time of patients with NSCLC

	Wald	df	*P*	Exp(B)	95.0% CI for Exp(B)	
					Lower	Upper
Gender (Male *vs.* Female)	1.371	1	0.242	1.369	0.809	2.316
Age (< 62 *vs.* ≥ 62)	0.141	1	0.707	0.904	0.534	1.531
Pathological grades (I + I–II + II *vs.* II–III + III)	0.381	1	0.537	0.827	0.451	1.514
AJCC stages (I *vs.* II + III)	12.320	1	0.000	2.898	1.600	5.248
CALR expression (Low *vs.* High)	5.153	1	0.023	0.521	0.297	0.915
PDIA3 expression (Low *vs.* High)	1.282	1	0.258	0.730	0.424	1.258

Cases of group “–” and “+” were assigned to group low. Cases of group “++” and “+++” were assigned to group high.

**Table 5:** Cox multivariate analysis of the correlation between clinicopathological parameters and survival time of patients with NSCLC (including combined expression of CALR and PDIA3)

	Wald	df	*P*	Exp(B)	95.0% CI for Exp(B)	
					Lower	Upper
Gender (Male *vs.* Female)	1.241	1	0.265	1.345	0.798	2.266
Age (< 62 *vs.* ≥ 62)	0.540	1	0.462	0.816	0.474	1.403
Pathological grades (I + I–II + II *vs.* II–III + III)	0.560	1	0.454	0.795	0.435	1.451
AJCC stages (I *vs.* II + III)	13.025	1	0.000	3.002	1.653	5.454
Combined expression of CALR and PDIA3						
Low PDIA3 and low CALR	12.148	3	0.007			
Low PDIA3 and high CALR	6.792	1	0.009	0.340	0.151	0.765
High PDIA3 and low CALR	3.202	1	0.074	0.427	0.168	1.085
High PDIA3 and high CALR	11.331	1	0.001	0.337	0.179	0.635

Cases of group “–” and “+” were assigned to group low. Cases of group “++” and “+++” were assigned to group high.

## DISCUSSION

Several researchers have devoted their studies to the risk of lung cancer, which indicate that tobacco use, environmental pollution, food, genetics, and chronic obstructive pulmonary disease are the main risks for lung cancer, whereas not enough attention has been paid to prevention and diagnosis of lung cancer [2]. It is of great importance to identify the potential specific biomarkers which perform an indispensable part in the prediction and diagnosis of lung cancer. In our study, we performed a quantitative proteomic analysis on 16 patients including NSCLC and paired adjacent non-tumor tissues using label-free quantitative proteomics and LC-MS/MS to identify differentially expressed proteins. Proteomic-based approaches carry out analyses mainly at translational levels and complex post-translational levels, which are not conducted by gene analysis [8]. These analyses at translational levels and complex post-translational levels perform an important role in detecting the complex cancer-related biological processes and potential prognostic biomarkers for cancers. Our results provided a potential biomarker dataset for NSCLC prognosis. Moreover, bioinformatics analyses showed cellular component, molecular function, biological process and signal transduction pathways of the dysregulated proteins. A more in-depth analysis by bioinformatics would be beneficial for further studies including more molecular biology studies and mechanism of NSCLC development.

The selected dysregulated proteins CALR and PDIA3 were found to be over-expressed in NSCLC compared with adjacent non-tumor lung tissues. Our data were in accordance with findings in breast cancer [9]. CALR is a major Ca^2+^-binding protein in the endoplasmic reticula (ER) lumen [10] and plays an important role in quality control processes during protein synthesis and folding [11] and the regulation of Ca^2+^ homeostasis and Ca^2+^ dependent pathways [12]. CALR also functions as an “eat me” signal and induces the immunogenic tumor cell death after translocation from the ER to the cytosol and the cell surface [13–15]. PDIA3 is a soluble glycoprotein and a member of the PDI family [16]. It participates in the assembly of major histocompatibility complex class I [17, 18] and catalyzes the disulfide oxidation, isomerization and reduction of native glycoproteins [19].

CALR has been reported as a potential biomarker in several types of cancer including neuroblastoma, gastric, bladder, esophageal, breast, esophageal cancer and acute myeloid leukemia [20–26]. PDIA3 is also involved in some types of tumors, such as breast, gastric, ovarian, hepatocellular, colorectal and laryngeal carcinoma [27–32]. However, the individual functions of CALR and PDIA3 are reported inconsistently in different tumors. For example, CALR overexpression enhances angiogenesis, and facilitates proliferation and migration of gastric cancer cells, which is in line with the association of CALR with tumor invasion, lymph node metastasis, and poor survival in gastric cancer patients [21]. CALR promotes migration and invasion of esophageal cancer cells by up-regulating neuropilin-1 expression via STAT5A and neuropilin-1 is a critical downstream effector of CALR in promoting cell migration and invasion [25]. On the contrary, high levels of CALR on the surface of malignant myeloblasts positively correlate with the ability of autologous T cells to secrete interferon-γ on stimulation with blast-derived dendritic cell, facilitating cellular anticancer immune responses in AML patients [26]. Similarly, PDIA3 expression is associated with tumor proliferation and decreases apoptosis in hepatocellular carcinoma, and increased expression of PDIA3 predicts poor prognosis [30]. Moreover, PDIA3 modulates radioresistance of laryngeal cancer cells by directly activating STAT3 and, in turn, triggers increased Mcl-1 expression, thereby contributing to tumor radioresistance of laryngeal cancer cells and poor outcomes in patients with laryngeal cancer in response to radiotherapy [32]. However, the presence of autoantibodies to PDIA3 antigen favors the development of an efficient and specific T-cell response against PDIA3 in patients with colorectal cancer, indicating that they have antitumor effector functions [31]. Therefore, we infer that CALR and PDIA3 exert a positive or negative effect on prognosis of patients with cancer by regulating the cancer cells themselves or the immune responses in the tumor microenvironment.

In NSCLC, CALR is described as a positive prognostic factor by increasing accumulation of antitumor immune cells [33]. The effect of PDIA3 on NSCLC has not been investigated. In our study, Kaplan-Meier analysis revealed that double low expressions of CALR and PDIA3 were positively associated with poor OS in NSCLC and double high expressions were positively associated with better OS in NSCLC, which indicated CALR and PDIA3 played positive roles in the prognosis of NSCLC. To understand the biological roles of overexpressed CALR and PDIA3 on NSCLC tumor cell itself, two plasmids containing CALR and PDIA3 genes were individually or jointly transfected into A549 and H460 cells. We found that the increased CALR or/and PDIA3 expressions were not significantly related to the cell proliferation, migration and invasion as revealed by CCK8 assay, scratch assay and cell invasion assay (data not shown). Therefore, we inferred from these findings that increased CALR and PDIA3 in NSCLC are considered as antigen markers of immunogenicity, which are able to mount an immune response, similar to PDIA3 in colorectal cancer [31], facilitating anticancer immunosurveillance and contributing to better clinical outcome. The detailed mechanism needs further study and investigation.

CALR is reported to be served as a chaperone interacting with PDIA3 via P-domain in colon cancer cells [19, 34]. However, there is no evidence to prove the relationship of CALR and PDIA3 in NSCLC. We found in our study that CALR expression was positively associated with PDIA3 expression in NSCLC, and both were interacted with each other. Cox regression analysis indicated that CALR, but not PDIA3 expression was an independent prognostic factor for patients with NSCLC. However, combined expression of CALR and PDIA3 was found to be an independent prognostic factor for patients with NSCLC and improved the accuracy of prediction. Some studies show that CALR and PDIA3 can translocate from the ER to the cell surface and facilitate tumor cell recognition and engulfment by dendritic cells and subsequent T-cell mediates elimination of the tumor, inducing the activation of adaptive immune responses in the tumor microenvironment [19, 35]. CALR exposure is controlled by PDIA3 exposure, and *vice versa*. They are translocated to the cell surface together in the same molecular complex. PDIA3 knockdown suppresses CALR exposure as well as phagocytosis by dendritic cells and abolishes the immunogenicity *in vivo*. Knockdown or the absence of CALR abolishes PDIA3 exposure [36]. These analyses suggest that combination of CALR and PDIA3 is more advantageous to enhance an immune response and contribute to better clinical outcome in patients with NSCLC, which is in line with Cox regression analysis that combined expression of CALR and PDIA3 was an efficient biomarker for the prognosis and more accurate in prediction of NSCLC prognosis compared with CALR and PDIA3 expression alone.

In conclusion, our results collectively provided a potential biomarker dataset for NSCLC prognosis and revealed that CALR and PDIA3 were over-expressed in NSCLC compared with adjacent non-tumor lung tissues. CALR and PDIA3 were co-localized and interacted with each other in NSCLC. Low expressions of CALR and PDIA3 were positively associated with poor OS. Combination of CALR and PDIA3 expressions may serve as an efficient biomarker and improve the prediction of NSCLC prognosis significantly.

## MATERIALS AND METHODS

### NSCLC and adjacent non-tumor lung tissues

A total of 16 paired samples of NSCLC and adjacent non-tumor lung tissues were collected from patients who underwent surgery between July 2009 and April 2010 and were preserved in the Department of Pathology (Department of Cell Biology, Fourth Military Medical University). Of the patients, 12.5%, 56.2% and 31.3% of cases were presented with the American Joint Committee on Cancer (AJCC) clinical stage I to III respectively.

### Mass spectrometry analyses

The tissues were treated with RIPA lysis buffer, and the protein samples were loaded to 10% SDS-PAGE gel. After coomassie brilliant blue staining, the gels were divided into five equal parts according to the distribution of molecular weight. Gel samples were evaporated using acetonitrile and reconstituted in 1% (v/v) formic acid, followed by trypsin digestion, prior to injection into the mass spectrometer (Model LTQ, Thermo Fisher Scientific, MA, USA). The mass spectrometer was calibrated using standard compounds and operated in the data-dependent mode in which the instrument cycled between full MS scans (m/z 300–2000) and the MS/MS data were collected by targeting MS/MS scans on the ten most abundant ions occurring in the MS scan. All raw files were analyzed using MaxQuant software (version 1.5.2.8) with Swiss-Prot human database (03/2016; 20,210 entries, www.uniprot.org). Carbamidomethylation of cysteine was selected as a fixed modification, while oxidation of methionine and N-terminal acetylation were selected as variable modifications. Mass tolerances for first search peptide and the main search peptide were set at 20 ppm and 4.5 ppm, respectively. Maximal false discovery rate for peptide spectral match and proteins was set to 0.01. Proteins were defined as differentially expressed if the ratios were > 1.5 or < 0.67 in NSCLC compared with adjacent non-tumor lung tissues with a significant change (*P* < 0.05). After label-free relative quantitation with IBAQ from MaxQuant, GO analysis, KEGG pathway analysis and IPA were conducted to analyze the protein-protein interaction network among the identified proteins by LC-MS/MS. The analyses were performed by Keecloud Biotech Co., Ltd. (Shanghai, China).

### Cell culture

The NSCLC cell line A549 was obtained from the American Type Culture Collection and H460 cell line was purchased from the Shanghai Institute for Biological Sciences (Shanghai, China). The cell lines have been tested and authenticated using Short Tandem Repeat DNA profiling by Beijing Microread Genetics Co., Ltd (Beijing, China) and were cultured at 37°C under 5% CO_2_ in RPMI 1640 medium supplemented with 10% fetal bovine serum, 1% penicillin/streptomycin and 2% L-glutamine.

### RNA extraction and real-time PCR analysis

The 20 paired NSCLC and adjacent non-tumor lung tissues samples for real-time PCR assay were collected from patients who underwent surgery in the Department of Thoracic Surgery (Tangdu hospital, Fourth Military Medical University). Total RNA was extracted from tissues using Total RNA Kit II (Omega Bio-tek, GA, USA) following manufacturer’s instructions, and then reversely transcribed to cDNA using the PrimeScript RT Reagent Kit (TaKaRa, Kusatsu, Japan). Real-time PCR was carried out using the SYBR Premix Ex Taq II Kit (TaKaRa, Kusatsu, Japan). The sequences of PCR primers were listed as follows:

Human CALR-F 5′-CGAGCCTTTCAGCAACA-3′,

Human CALR-R 5′-CAGACTTGACCTGCCAGAG-3′;

Human PDIA3-F 5′-GCCTCCGACGTGCTAGAAC-3′,

Human PDIA3-R 5′-GCGAAGAACTCGACGAGCAT-3′;

Human GAPDH-F 5′-GCACCGTCAAGGCTGAGAAC-3′,

Human GAPDH-R 5′-TGGTGAAGACGCCAGTGGA-3′.

### Western blot analysis

NSCLC tissue (Department of Thoracic Surgery, Tangdu hospital, Fourth Military Medical University) and cell were treated with RIPA lysis buffer containing phenylmethylsulfonyl fluoride, protease inhibitors and phosphatase inhibitors (KeyGen BioTech, Nanjing, China). The BCA Protein Assay Kit (Thermo Fisher Scientific, MA, USA) was employed to quantify amounts of protein. After boiling for 10 minutes, equal amounts of protein were loaded to 10% SDS-PAGE gel and then transferred to PVDF membranes (Millipore, Boston, MA, USA). After blocking with 5% non-fat milk for 1 hour, the membrane was incubated with the designated primary antibodies (anti-CALR, sc-166837, Santa Cruz, CA, USA, dilution 1:500; anti-PDIA3, sc-23886, Santa Cruz, CA, USA, dilution 1:500; anti-GAPDH, R1210-1, HuaAn, Hangzhou, China, dilution 1:2000) at room temperature for 3 hours. The images were developed after incubation with the secondary antibodies (goat anti-mouse IgG(H+L) antibody, 31430, Thermo Fisher Scientific, MA, USA, dilution 1:5000; goat anti-rabbit IgG(H+L) antibody, 31460, Thermo Fisher Scientific, MA, USA, dilution 1:5000) at room temperature for 1 hour.

### Immunohistochemistry analysis

Immunohistochemistry was performed using two serial tissue microarray (TMA) of NSCLC purchased from Shanghai Biochip Company (Shanghai, China). TMA contained 87 paired NSCLC and adjacent non-tumor lung tissues and 6 NSCLC tissues. Paraffin section was dewaxed, followed by antigen retrieval with 10 μmol/L citrate buffer at pH6.0. Deparaffinized sections were treated with methanol containing 3% hydrogen peroxide for 15 minutes. After washing with PBS, sections were incubated with blocking serum for 30 minutes. Then, sections were incubated with anti-CALR (sc-166837, Santa Cruz, CA, USA) and anti-PDIA3 (15967-1-AP, Proteintech, IL, USA) at 4°C overnight. Following incubation, immunoperoxidase staining was conducted using a streptavidin-peroxidase kit (Zhongshan Jinqiao Co., Beijing, China) and treated with 3,3′-diaminobenzidine (Zhongshan Jinqiao Co., Beijing, China) to detect the target proteins. Hematoxylin was used to counterstain the nuclei. The original overview scannings of tissue microarray were shown in Supplementary Figure 7.

### Immunohistochemistry evaluation

The TMA sections were evaluated by two independent pathologists, who were blinded to the experiment. The intensity and density of positive cells were two important evaluation parameters in the scoring. The intensity of positive cells was evaluated by the color of the positive cells, which was classified as 0 (no staining), 1 (weak), 2 (moderate) and 3 (strong). The density of positive cells was valued into four levels: 0 (staining ≤ 5%), 1 (5% < staining ≤ 30%), 2 (30% < staining ≤ 70%) and 3 (staining > 70%). According to the total scores summed by the scores of the intensity and density of positive cells, the levels of staining were graded as “-” (score 0–1), “+” (score 2–3), “++” (score 4–5) and “+++” (score 6). Among the 93 NSCLC samples, 5 samples were excluded from the study owing to absence of information of TNM phase (4 cases) and tumor necrosis (1 case). Finally 88 NSCLC samples were used for immunohistochemistry analysis. The patient characteristics were shown in Table 6.

**Table 6:** Patient characteristics

Parameters	*n*
Gender	
Male	46
Female	42
Age (years)	
Mean, media (range)	62.0, 61.5 (30–84)
Tumor sizes (cm)	
≤ 3	33
> 3	55
Survival status	
Survival	21
Death	67
Survival time (months)	
Mean, media (range)	44.1, 39.0 (2–121)
Lymph node metastases	
0	37
≥ 1	51
AJCC stages	
I	27
II + III	61
Pathological grades	
I + I–II + II	62
II–III + III	26

### Immunofluorescence assay

The cells were cultured on small round cover glass, fixed with 4% paraformaldehyde, permeabilized with 0.2% Triton X-100, blocked with goat serum, and then treated with anti-PDIA3 (sc-23886, Santa Cruz, CA, USA,) and anti-CALR (10292-1-AP, Proteintech, IL, USA). After incubation with the fluorescence labeling secondary antibodies (A21202, Thermo Fisher Scientific, MA, USA; A31572, Thermo Fisher Scientific, MA, USA), the co-localization expression was visualized under a confocal florescence microscopy (Nikon, Japan).

### Co-immunoprecipitation (co-IP)

Co-IP was performed using a Pierce™ co-IP Kit (Thermo Fisher Scientific, MA, USA) according to the manufacturer’s protocol. Cell lysates of A549 and H460 were prepared, and 10 μg of affinity-purified antibody (anti-CALR, sc-166837, Santa Cruz, CA, USA; anti-PDIA3, sc-23886, Santa Cruz, CA, USA) of each co-IP assay was used for antibody immobilization. After pre-clear cell lysates using the control agarose resin, cell lysates were incubated with gentle mixing or rocking overnight at 4°C. The eluted samples were analyzed by western blot after elution of columns.

### Statistical analysis

All the statistical analyses were performed using SPSS 19.0 (SPSS, Inc., Chicago, IL, USA). Significant differences of molecule levels between NSCLC and adjacent non-tumor lung tissues were analyzed by Mann-Whitney *U* tests with a two-tailed distribution. The correlations of CALR and PDIA3 expressions with clinicopathological parameters of patients were analyzed by Pearson’s chi-squared test (χ^2^). Spearman correlation analysis was used to analyze the correlation between CALR and PDIA3 expressions. Overall survival (OS) was calculated using Kaplan-Meier analysis and log-rank test. Multivariate survival analysis was conducted by using the Cox regression models. *P* < 0.05 was considered to be significant.

## SUPPLEMENTARY MATERIALS FIGURES AND TABLE




