# Supplementary material for: Combination of CALR and PDIA3 is a potential prognostic biomarker for non-small cell lung cancer
Source: Oncotarget. 2017 Jun 16;8(57):96945–57. doi: 10.18632/oncotarget.18547 (PMC5722536; doi:10.18632/oncotarget.18547)
Supplement: Supplementary file 1 [file oncotarget-08-96945-s001.pdf]

Combination of CALR and PDIA3 is a potential prognostic biomarker for non-small cell lung cancer

Supplementary Materials

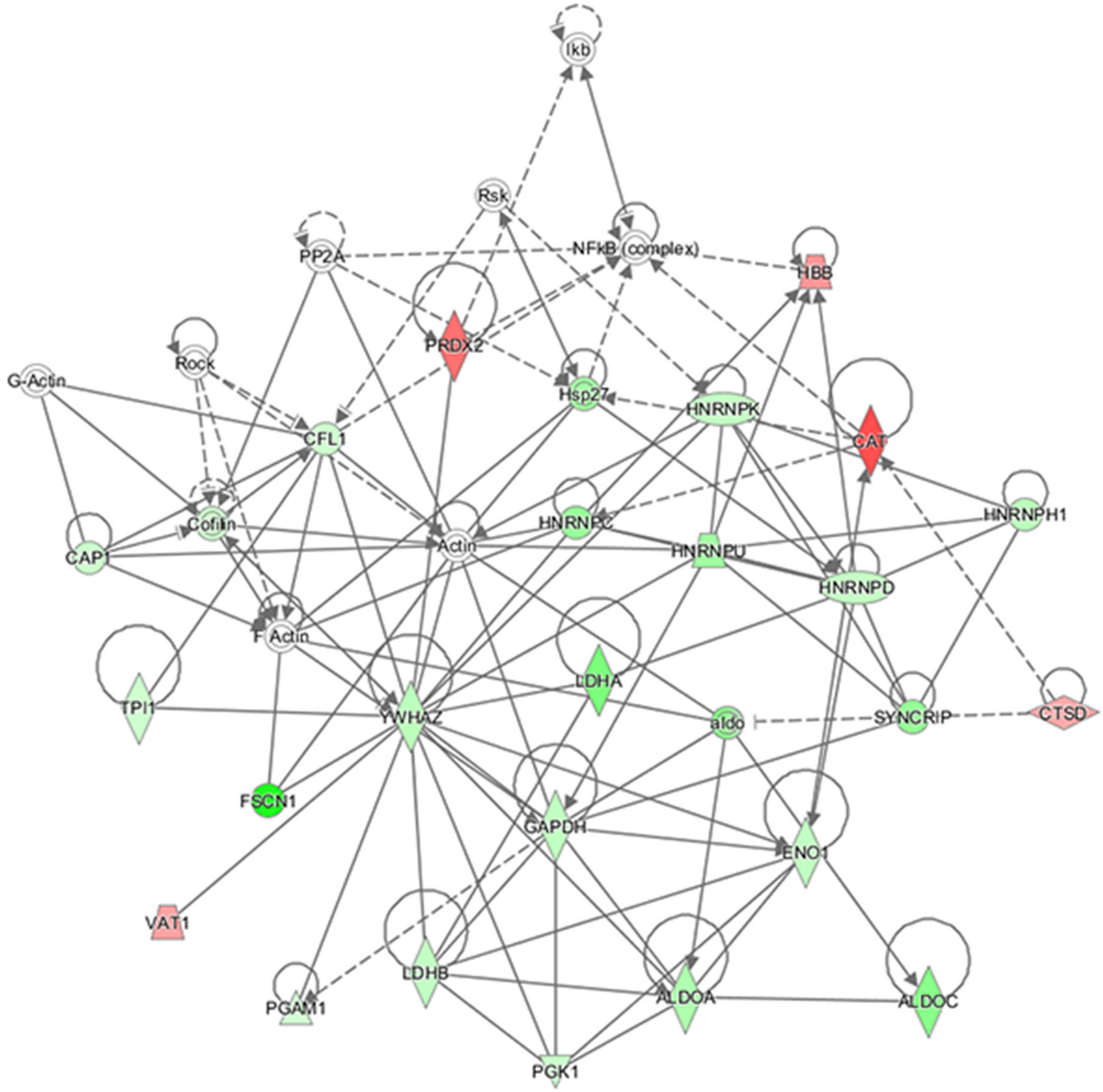

Supplementary Figure 1: Network analysis of the dysregulated proteins by IPA. Network: Carbohydrate metabolism/cellular movement/hematological disease.

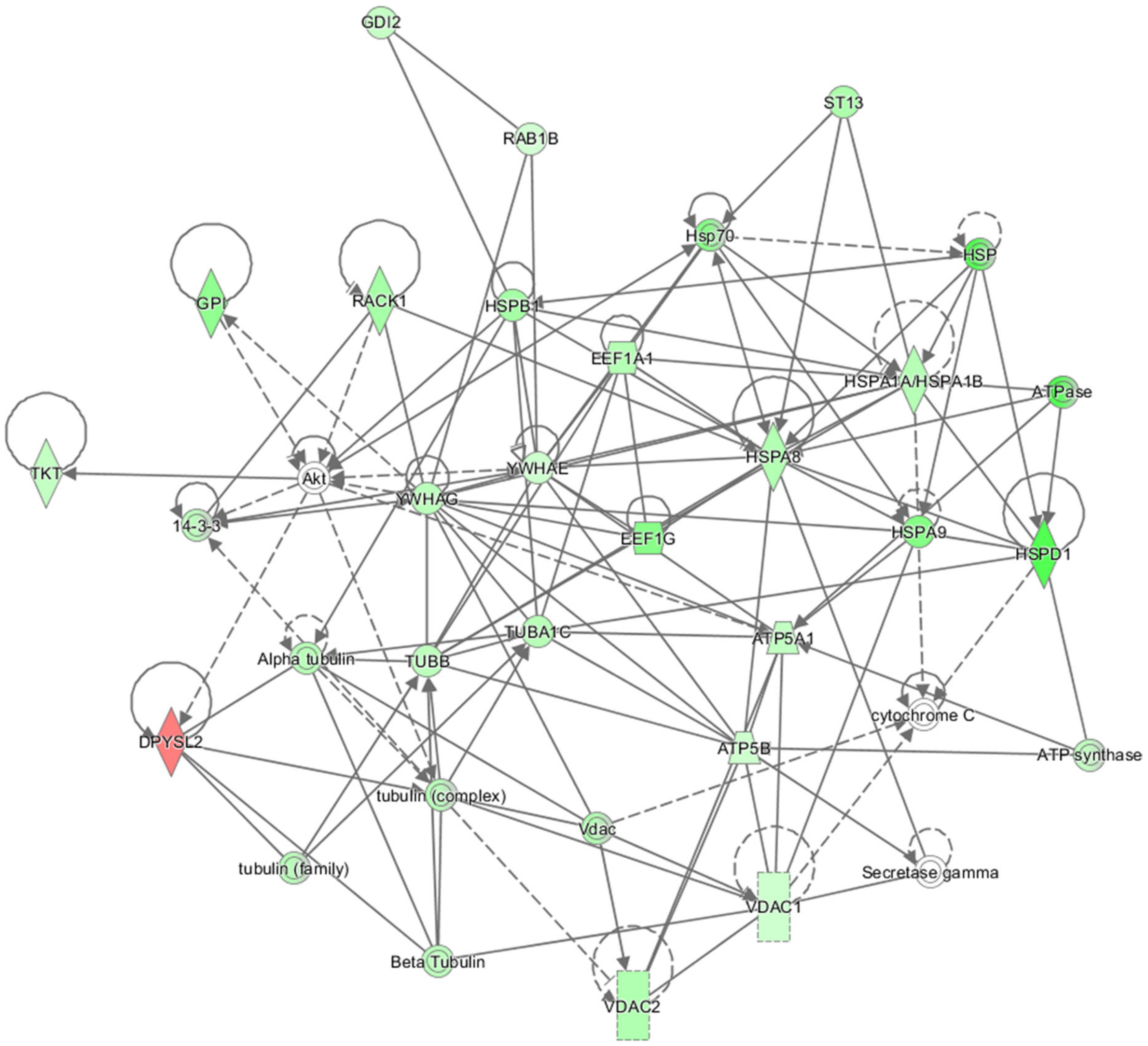

Supplementary Figure 2: Network analysis of the dysregulated proteins by IPA. Network: Post-translational modification/ protein folding/nucleic acid metabolism.

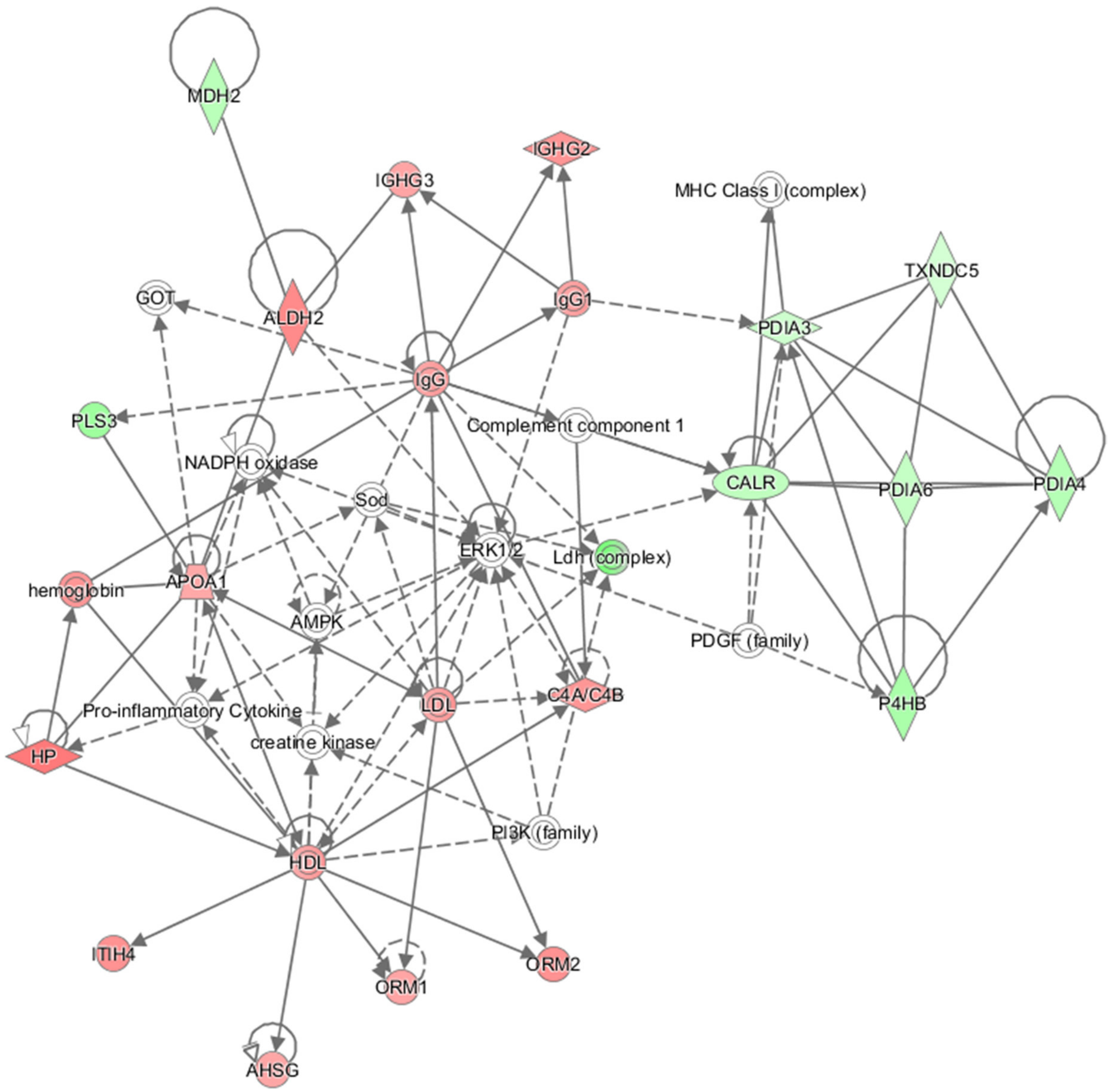

Supplementary Figure 3: Network analysis of the dysregulated proteins by IPA. Network: Endocrine system disorders/ organismal injury and abnormalities/neurological disease.

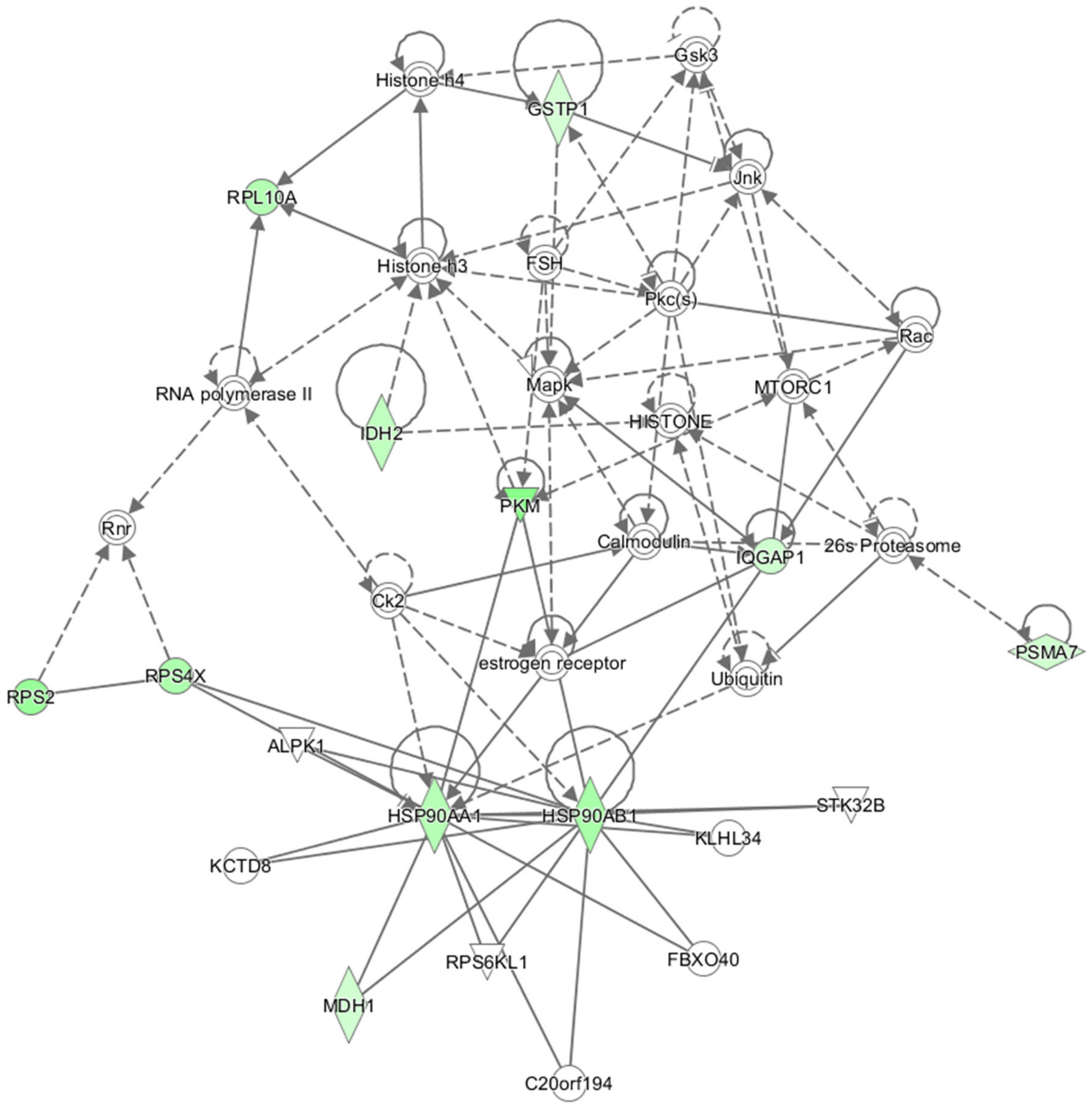

Supplementary Figure 4: Network analysis of the dysregulated proteins by IPA. Network: Cancer/gastrointestinal disease/hepatic system disease.

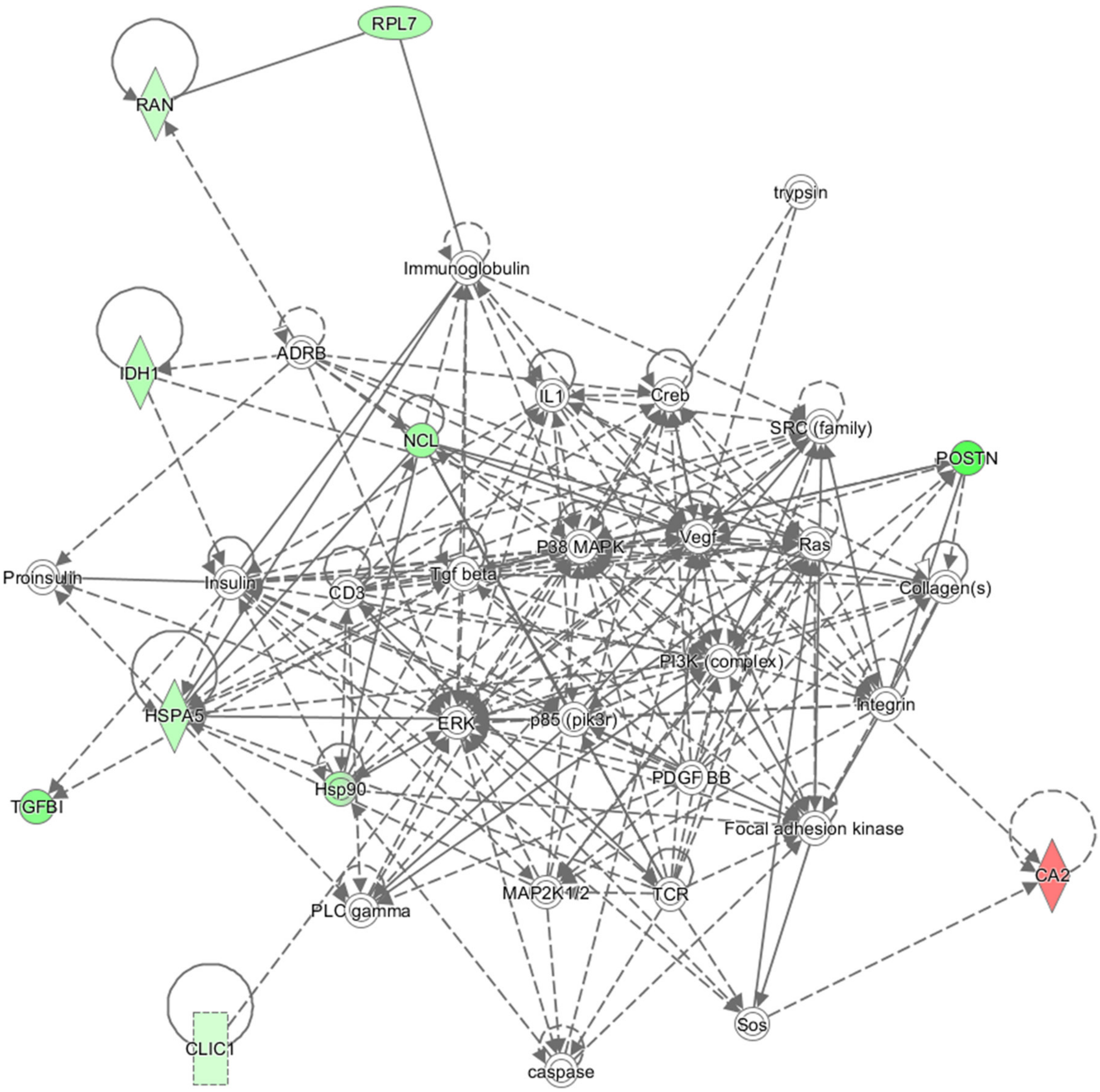

Supplementary Figure 5: Network analysis of the dysregulated proteins by IPA. Network: Connective tissue disorders/ developmental disorder/hereditary disorder.

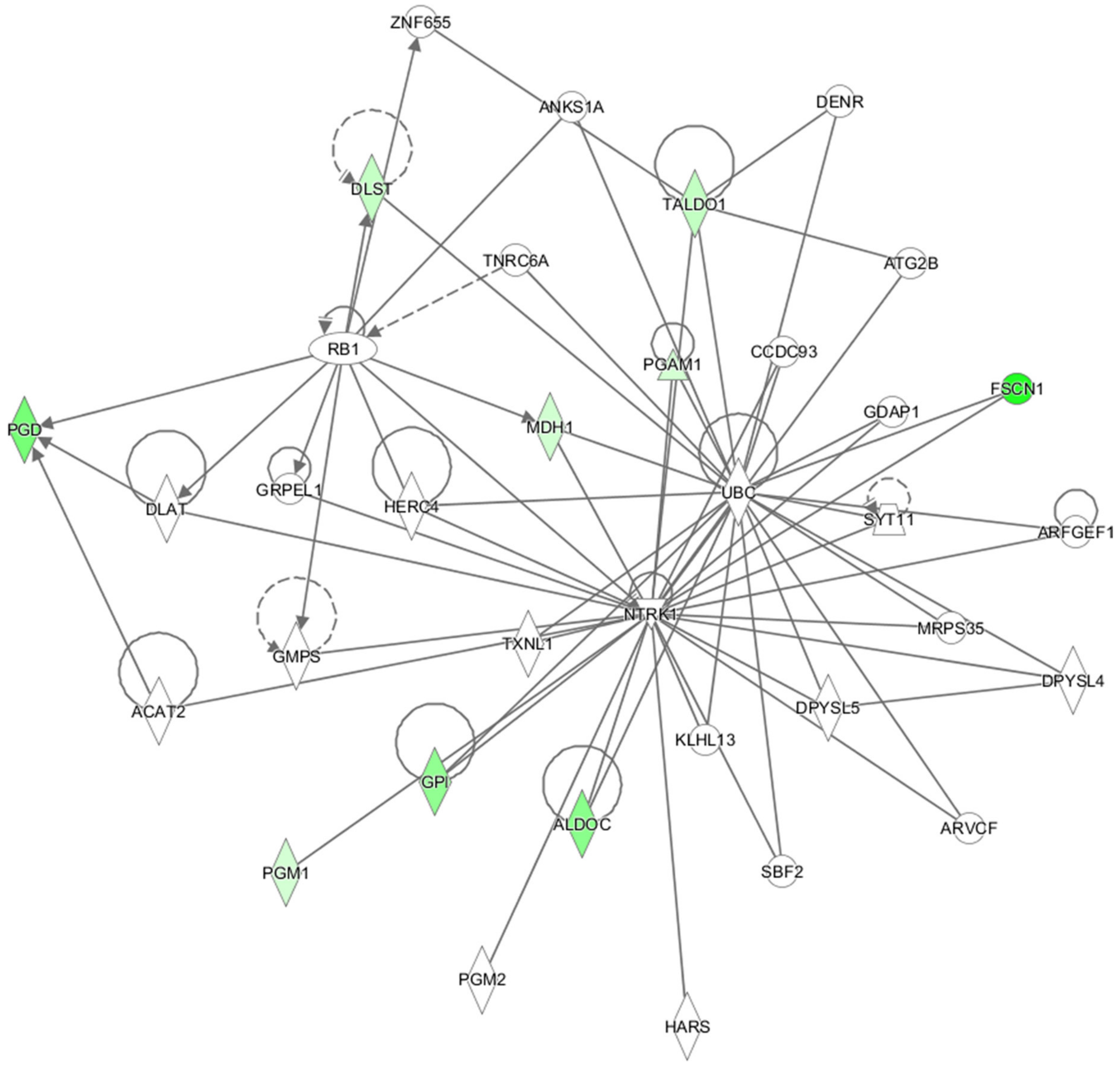

Supplementary Figure 6: Network analysis of the dysregulated proteins by IPA. Network: Carbohydrate metabolism/nucleic acid metabolism/small molecule biochemistry.

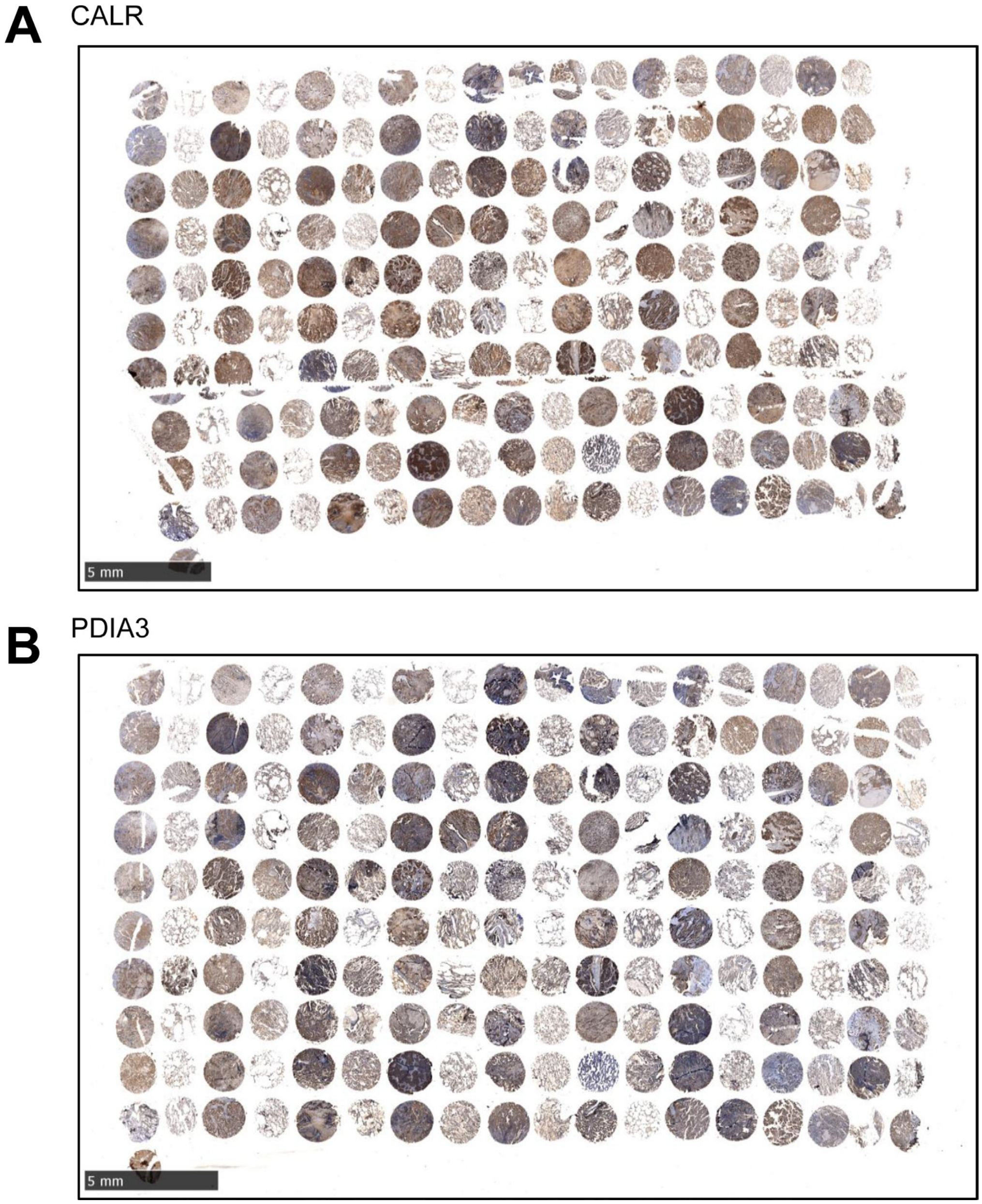

Supplementary Figure 7: The original overview scanning of tissue microarray. (**A**) The original overview scanning of tissue microarray for CALR. Scale bar = 5 mm. (**B**) The original overview scanning of tissue microarray for PDIA3. Scale bar = 5 mm.

Supplementary Table 1: Differentially expressed proteins between NSCLC (T) and adjacent non-tumor lung tissues (N) by LC-MS/MS. See Supplementary_Table_1
