# Supplementary material for: Combination of CALR and PDIA3 is a potential prognostic biomarker for non-small cell lung cancer
Source: Oncotarget. 2017 Jun 16;8(57):96945–57. doi: 10.18632/oncotarget.18547 (PMC5722536; doi:10.18632/oncotarget.18547)
Supplement: Supplementary file 2 [file oncotarget-08-96945-s002.docx]

**Table S1.** Differentially expressed proteins between NSCLC (T) and adjacent non-tumor lung tissues (N) by LC-MS/MS

| Protein IDs | Log_2_(ratio (N/T)) | -Log_10_(p-value) |
| --- | --- | --- |
| sp\|O00299\|CLIC1_HUMAN | -0.630929 | 3.290255 |
| sp\|O14818\|PSA7_HUMAN;  sp\|Q8TAA3\|PSA7L_HUMAN | -0.757054 | 4.308787 |
| sp\|O60506\|HNRPQ_HUMAN | -1.899719 | 5.97181 |
| sp\|O75874\|IDHC_HUMAN | -1.388396 | 5.329465 |
| sp\|P00338\|LDHA_HUMAN | -2.209723 | 16.27716 |
| sp\|P00558\|PGK1_HUMAN | -0.831604 | 6.223465 |
| sp\|P00738\|HPT_HUMAN | 1.378255 | 3.184068 |
| sp\|P00915\|CAH1_HUMAN | 2.013343 | 9.680778 |
| sp\|P00918\|CAH2_HUMAN | 1.385734 | 5.975212 |
| sp\|P01623\|KV305_HUMAN | 0.596626 | 1.947411 |
| sp\|P01859\|IGHG2_HUMAN | 1.063599 | 2.399739 |
| sp\|P01860\|IGHG3_HUMAN | 0.822874 | 1.417422 |
| sp\|P02647\|APOA1_HUMAN | 0.755914 | 3.697417 |
| sp\|P02763\|A1AG1_HUMAN | 0.818113 | 2.774781 |
| sp\|P02765\|FETUA_HUMAN | 0.798142 | 2.625927 |
| sp\|P04040\|CATA_HUMAN | 1.791827 | 6.419615 |
| sp\|P04075\|ALDOA_HUMAN | -1.513906 | 10.18891 |
| sp\|P04406\|G3P_HUMAN | -1.113208 | 8.867123 |
| sp\|P04792\|HSPB1_HUMAN | -1.667277 | 3.529818 |
| sp\|P05091\|ALDH2_HUMAN | 1.195221 | 4.180473 |
| sp\|P06576\|ATPB_HUMAN | -0.812183 | 2.648581 |
| sp\|P06733\|ENOA_HUMAN | -1.107568 | 5.804892 |
| sp\|P06744\|G6PI_HUMAN;  sp\|Q8N196\|SIX5_HUMAN | -1.965392 | 7.728942 |
| sp\|P07195\|LDHB_HUMAN | -1.094115 | 7.079098 |
| sp\|P07237\|PDIA1_HUMAN | -1.585611 | 5.880245 |
| sp\|P07339\|CATD_HUMAN | 0.733876 | 2.159658 |
| sp\|P07437\|TBB5_HUMAN;  sp\|A6NNZ2\|TBB8L_HUMAN;  sp\|Q9H4B7\|TBB1_HUMAN | -1.217646 | 3.059658 |
| sp\|P07900\|HS90A_HUMAN;  sp\|Q14568\|HS902_HUMAN;  sp\|Q58FG0\|HS905_HUMAN | -1.355833 | 5.718456 |
| sp\|P07910\|HNRPC_HUMAN;  sp\|B2RXH8\|HNRC2_HUMAN | -1.837952 | 11.00876 |
| sp\|P08238\|HS90B_HUMAN | -1.600817 | 6.974453 |
| sp\|P09211\|GSTP1_HUMAN | -0.592337 | 1.722074 |
| sp\|P09972\|ALDOC_HUMAN | -2.04762 | 12.64433 |
| sp\|P0C0L4\|CO4A_HUMAN | 1.028721 | 2.747564 |
| sp\|P0DMV9\|HS71B_HUMAN;  sp\|P0DMV8\|HS71A_HUMAN | -1.35642 | 3.528101 |
| sp\|P10809\|CH60_HUMAN | -2.629181 | 11.68656 |
| sp\|P11021\|GRP78_HUMAN | -1.252084 | 4.717889 |
| sp\|P11142\|HSP7C_HUMAN | -1.43631 | 5.632693 |
| sp\|P13667\|PDIA4_HUMAN | -1.370529 | 2.802317 |
| sp\|P13797\|PLST_HUMAN | -1.769691 | 5.737296 |
| sp\|P14618\|KPYM_HUMAN | -2.061129 | 8.426016 |
| sp\|P15880\|RS2_HUMAN | -1.844887 | 8.309552 |
| sp\|P18124\|RL7_HUMAN | -1.526337 | 7.093367 |
| sp\|P18669\|PGAM1_HUMAN;  sp\|P15259\|PGAM2_HUMAN;  sp\|Q8N0Y7\|PGAM4_HUMAN | -0.624349 | 4.126474 |
| sp\|P19338\|NUCL_HUMAN | -1.784182 | 7.663018 |
| sp\|P19652\|A1AG2_HUMAN | 1.103595 | 3.992572 |
| sp\|P21796\|VDAC1_HUMAN | -0.794461 | 4.231446 |
| sp\|P23528\|COF1_HUMAN | -0.772135 | 4.180746 |
| sp\|P25705\|ATPA_HUMAN | -1.125269 | 4.772286 |
| sp\|P26641\|EF1G_HUMAN | -2.077522 | 10.55068 |
| sp\|P27797\|CALR_HUMAN | -1.135595 | 3.864496 |
| sp\|P29401\|TKT_HUMAN;  sp\|P51854\|TKTL1_HUMAN | -1.068609 | 2.903595 |
| sp\|P30101\|PDIA3_HUMAN | -0.796017 | 2.557509 |
| sp\|P31943\|HNRH1_HUMAN | -1.28185 | 6.045726 |
| sp\|P32119\|PRDX2_HUMAN | 1.474202 | 8.237599 |
| sp\|P36871\|PGM1_HUMAN | -0.609827 | 1.968532 |
| sp\|P36957\|ODO2_HUMAN | -1.010192 | 2.378234 |
| sp\|P37837\|TALDO_HUMAN | -1.094906 | 3.077536 |
| sp\|P38646\|GRP75_HUMAN | -2.124062 | 5.553662 |
| sp\|P40925\|MDHC_HUMAN | -0.687346 | 3.43095 |
| sp\|P40926\|MDHM_HUMAN | -1.302451 | 7.481512 |
| sp\|P45880\|VDAC2_HUMAN | -1.479413 | 9.765341 |
| sp\|P46940\|IQGA1_HUMAN | -0.765468 | 1.721902 |
| sp\|P48735\|IDHP_HUMAN | -1.131802 | 6.501914 |
| sp\|P50395\|GDIB_HUMAN | -1.001827 | 3.379765 |
| sp\|P50502\|F10A1_HUMAN;  sp\|Q8NFI4\|F10A5_HUMAN;  sp\|Q8IZP2\|ST134_HUMAN | -1.538599 | 4.274824 |
| sp\|P52209\|6PGD_HUMAN | -2.274511 | 10.42976 |
| sp\|P60174\|TPIS_HUMAN | -0.756897 | 3.792488 |
| sp\|P61978\|HNRPK_HUMAN | -1.046012 | 6.206665 |
| sp\|P61981\|1433G_HUMAN | -1.245674 | 6.308288 |
| sp\|P62258\|1433E_HUMAN | -0.787309 | 3.517155 |
| sp\|P62701\|RS4X_HUMAN;  sp\|Q8TD47\|RS4Y2_HUMAN;  sp\|P22090\|RS4Y1_HUMAN | -1.525254 | 9.453171 |
| sp\|P62826\|RAN_HUMAN | -1.005185 | 4.207941 |
| sp\|P62906\|RL10A_HUMAN | -1.402582 | 7.949205 |
| sp\|P63104\|1433Z_HUMAN | -1.252265 | 5.97721 |
| sp\|P63244\|RACK1_HUMAN | -1.647362 | 9.371501 |
| sp\|P68104\|EF1A1_HUMAN;  sp\|Q5VTE0\|EF1A3_HUMAN | -1.398287 | 4.378182 |
| sp\|P68871\|HBB_HUMAN | 1.03658 | 4.371514 |
| sp\|P69905\|HBA_HUMAN | 0.864846 | 3.643789 |
| sp\|Q00839\|HNRPU_HUMAN | -1.708794 | 6.884285 |
| sp\|Q01518\|CAP1_HUMAN | -0.818093 | 2.2207 |
| sp\|Q14103\|HNRPD_HUMAN | -1.035154 | 5.099608 |
| sp\|Q14624\|ITIH4_HUMAN | 1.104642 | 5.563547 |
| sp\|Q15063\|POSTN_HUMAN | -2.584978 | 10.27723 |
| sp\|Q15084\|PDIA6_HUMAN | -1.037059 | 3.355738 |
| sp\|Q15582\|BGH3_HUMAN | -2.081086 | 6.961131 |
| sp\|Q16555\|DPYL2_HUMAN | 1.339655 | 7.54857 |
| sp\|Q16658\|FSCN1_HUMAN | -2.998538 | 11.65245 |
| sp\|Q8NBS9\|TXND5_HUMAN | -0.619511 | 1.881887 |
| sp\|Q99536\|VAT1_HUMAN | 0.844854 | 2.580345 |
| sp\|Q9BQE3\|TBA1C_HUMAN | -1.320334 | 3.352781 |
| sp\|Q9H0U4\|RAB1B_HUMAN;  sp\|Q92928\|RAB1C_HUMAN;  sp\|P59190\|RAB15_HUMAN | -0.621662 | 2.143577 |
